# Dataset of Ukrainian migrant workers opinions on their stay in Poland during COVID-19 lockdown

**DOI:** 10.1016/j.dib.2021.107415

**Published:** 2021-09-22

**Authors:** Dorota Kowalewska, Anita Adamczyk, Monika Trojanowska–Strzęboszewska

**Affiliations:** a University of Szczecin, Poland; b Adam Mickiewicz University Poznań, Poland; c Cardinal Stefan Wyszyński University (UKSW), Poland

**Keywords:** Migrant workers, Ukrainians, Poland, Immigration, COVID-19, Lockdown

## Abstract

The study contains a dataset from survey on the opinions of labour migrants from Ukraine in Poland, collected at the beginning of the COVID-19 pandemic. Participants (conducted in May and June 2020) were 617 migrant workers from Ukraine who remained in Poland during the first period of the pandemic in Poland. Due to limitations in face-to-face contact, the survey was conducted online (Google Forms questionnaire). The developed questionnaire was available in three languages: Ukrainian, Polish and Russian. The researchers were supported in sending the questionnaire by NGOs, Ukrainian minority organisations, as well as labour migrants from Ukraine who had participated in previous research conducted by the team.

The questionnaire contained 34 questions (in three languages: Ukrainian, Polish and Russian), including attribute variables of the participants). Questions addressed issues such as perception of COVID risks, changes in the respondent's labor market situation, and assessment of their quality-of-life changes.

The study will contribute to the knowledge of institutions and NGOs that work with labour migrants. The data collected provides a starting point for comparing the situation of migrant workers one year after the pandemic. The results of the study can be taken into account when planning migration management in the host country.

## Specifications Table

**Table:** 

Subject	Social Sciences
Specific subject area	Situation of labour migrants from Ukraine in the first COVID-19 lockdown period in Poland
Type of data	Aggregated tables, raw data in .xls format
How data were acquired	Survey
Data format	Raw
Parameters for data collection	The participants of the survey were 617 Ukrainian migrant workers who have remained in Poland during first COVID-19 lockdown in Poland. Due to epidemic restrictions, an online survey tool was used.
Description of data collection	The study was conducted in May and June 2020 using an online survey (Google Forms questionnaire). The research team developed a set of questions in three languages: Ukrainian, Polish and Russian. Since the online migrant survey required the credibility of the researchers in the study group, the snowball sampling and anonymous questionnaire were applied. Online distribution was supported initially by NGOs working with migrants, Ukrainian minority organizations in Poland, and migrants who had previously participated in research conducted by the team. Since the participation was self-selected, sample could not be weighted. During the post-implementation control, 62 questionnaires that were filled out incorrectly or incompletely were discarded. The questionnaire and raw data were translated into English.
Data source location	Country: Poland
Data accessibility	Repository name: Mendeley Direct URL to data: http://dx.doi.org/10.17632/3f3kvnsbdz.1

## Value of the Data

• The survey contributes to the expertise of institutions and NGOs that work with labour migrants. The pandemic situation increases the amount of information that migrants had to understand both in the area of work and the possibility to get help. It is important to notice the problems of people who, due to insufficient knowledge of the language of the host country, did not understand all the safety information and lockdown measures.
• The dataset concerns labor migrants' obtaining information about restrictions or seeking information about possible support from the host country. The results can be important for both NGOs and social support institutions about how, where, and in what language (whether migrants' language is relevant or whether they rather get information from sites in the language of the host country) to post relevant information.
• The data provides a starting point for comparing the situation of migrant workers one year after the pandemic. It should be noted that more pandemics are possible in the future, so the results of the study can be taken into account when planning migration management in the host country.

## 1 Data Description

From mid-March 2020, restrictions have been introduced in relation to the epidemiological threat. The aim of the survey was to identify the difficulties faced by Ukrainian citizens who have stayed in Poland.

The survey was provided by research team from following Polish scientific institutions: Institute of Political Science and Security Studies at the University of Szczecin, the Faculty of Political Science and Journalism at the Adam Mickiewicz University, Poznań and the Institute of Political Science and Administration at the Cardinal Stefan Wyszyński University, Warsaw.

In the description and tables below, we only present summaries of quantitative data. Text responses to the two open-ended questions is available in the data file (dataset and the questionnaire – translated to English - can be found in the attached link to Mendeley data repository).

The survey involved 617 respondents aged 19 to 79, with a significant majority of women (71.3%). More than half of the respondents have been living in Poland for more than 2 years (after adding up the answers ``2-5 years'' and ``more than 5 years''). Mazowieckie and Zachodniopomorskie voivodships were the most represented in the sample, but this over-representation should not affect further analysis of the situation of migrants during the pandemic, as in the surveyed period, different rules and restrictions for different regions had not yet been introduced (the same regulations applied throughout Poland). Respondents living in large cities were dominant (34.2% live in a city of 100,000 to 500,000 residents, and 27.4% resided in a city of more than half a million residents) (see Table 1 Socio-demographic variables).

**Table 1:** Socio-demographic variables.

	Freq (n)	Percent
**Gender**		
Male	177	28.7
Female	440	71.3
**Age**		
19 to 29	122	19.8
30 to 39	166	26.9
40 to 49	161	26.1
50 to 59	121	19.6
60 and more	13	2.1
*Missing*	*34*	*5.5*
Median	40.00	
Mean	39.70	
Std dev	11.08	
**Length of stay in Poland**		
Up to 4 months	23	3.7
From 4 to 6 months	51	8.3
More than 6 months but less than 1 year	62	10.0
1-2 years (also with breaks)	127	20.6
2-5 years (also with breaks)	251	40.7
More than 5 years (also with breaks)	103	16.7
**Residence status in Poland**		
A temporary residence permit for the purpose of performing work by a foreigner delegated by a foreign employer to work in Poland	9	1.5
A temporary residence permit for the purpose of performing work requiring high qualifications	19	3.1
I do not know	39	6.3
Long-term resident's EU residence permit	10	1.6
Permanent residence permit	40	6.5
Temporary residence and work permit	279	45.2
Temporary residence permit	153	24.8
Temporary residence permit for family members of Polish citizens and family members of foreigners	19	3.1
Temporary residence permit for the purpose of performing work for the purpose of conducting business activity	3	.5
Temporary residence permit in order to perform work in order to study at a university	32	5.2
*Missing*	*14*	*2.3*
**Number of people included in household (Poland)**		
I live alone	184	29.8
2 persons	229	37.1
3 persons	102	16.5
4 persons	71	11.5
5 persons	15	2.4
*Missing*	*16*	*2.6*
**Place of stay in Poland**		
A village away from the big city	12	1.9
A village near a big city	57	9.2
City up to 10,000 residents	52	8.4
City from 10,000 up to 100,000 residents	116	18.8
City from 100,000 to 500,000 residents	211	34.2
City over 500,000 residents	169	27.4
**The province in which respondents were staying during the research**		
Dolnośląskie	27	4.4
Kujawsko-Pomorskie	8	1.3
Łódzkie	23	3.7
Lubelskie	14	2.3
Lubuskie	19	3.1
Małopolskie	34	5.5
Mazowieckie	101	16.4
Opolskie	17	2.8
Podkarpackie	4	.6
Podlaskie	2	.3
Pomorskie	72	11.7
Śląskie	32	5.2
Świętokrzyskie	1	.2
Warmińsko-Mazurskie	5	.8
Wielkopolskie	55	8.9
Zachodniopomorskie	203	32.9

The vast majority of respondents believe that staying in Poland during the pandemic was a good choice (See Table 2). Among the costs associated with staying in the country, the respondents mention above all the fear of relatives' illness (45.9% of all cases), or their own illness (18.2%) (See Table 3 with multiple answers). 49.1% of respondents declared that their economic situation deteriorated during the pandemic, 47.5% that it did not change. 9.1% declared the pandemic forced them to apply for social care benefits (this level remains unchanged as 11.8% say they received benefits before the pandemic). The most frequently mentioned reasons for the deterioration of respondent situation were: reduced salaries, food price increases, loss of the job (see Table 5).

**Table 2:** Respondents’ opinions if staying in Poland during the pandemic was a good choice.

	Freq (n)	Percent
Definitely not	3	.5
Probably not	2	.3
Neither yes nor no	34	5.5
Probably yes	113	18.3
Definitely yes	460	74.6
Missing	5	.8
Total	617	100.0

**Table 3:** Main fears related with pandemic (multiple answer possible).

	N	% of responses	% of cases
Covid19 of my relatives/friends	283	22.1	45.9
Deterioration of living conditions	58	4.5	9.4
I have no concerns about the pandemic	76	5.9	12.3
I would get Covid	112	8.7	18.2
It will not be possible to go to Ukraine	189	14.7	30.6
Loss of a job	224	17.5	36.3
Reduced salary	110	8.6	17.8
The deepening economic crisis	230	17.9	37.3
Total	1282	100.0	207.8

**Table 4:** Opinions on changes respondent's material status in time of Covid pandemic.

	Freq (n)	%
It Got worse	303	49.1
It remained unchanged	293	47.5
Get better	21	3.4
Total	617	100.0

**Table 5:** Reasons for worsening respondent's economic situation.

	N	% of responses	% of cases
I lost my job	86	18.7	28.4
Increased food prices	89	19.4	29.4
Increased rental prices (apartment)	35	7.6	11.6
Loss of employment of a family member residing in Poland	43	9.4	14.2
Reduction of wages	169	36.8	55.8
Another answer	37	8.1	12.2
Total	459	100.0	151.5

According to the respondents the most common reasons for staying in Poland were having a job (59.2%) and having plans to live in Poland (45.2%). For 23.7% the reason for staying in Poland was better (than in Ukraine) medical care (see Table 6). The decision of the Polish authorities to close the borders was assessed positively by the migrant workers surveyed. The most frequently identified source of information about regulations during a pandemic by respondents was their employer and websites (Facebook included) (see Table 7).

**Table 6:** Reasons for choosing to stay in Poland during the pandemic: (multiple answers).

	N	% of responses	% of cases
Because I'm studying (in Poland)	51	3.5	8.3
Because of the job I have	365	24.8	59.2
Due to better medical care in Poland	146	9.9	23.7
Due to higher earnings in Poland	106	7.2	17.2
Due to the children attending school (in Poland)	68	4.6	11.0
Due to the situation of a family member (other than work)	11	0.7	1.8
Due to the work of a family member in Poland	81	5.5	13.1
For fear of forced quarantine in Ukraine	45	3.1	7.3
I did not know when it will be possible to return to Poland	118	8.0	19.1
I have tied my life plans with Poland and I do not intend to leave here	279	19.0	45.2
I was afraid (in general)	150	10.2	24.3
I was afraid of a compulsory quarantine in Ukraine	4	0.3	0.6
I was afraid that the situation in Ukraine during the pandemic would be worse than in Poland	22	1.5	3.6
Another reason	23	1.6	3.7
Total	1469	100.0	238.1

**Table 7:** Respondents’ answer on question about how they heard about the measures during a pandemic (multiple answers).

	N	% of responses	% of cases
From conversations with priests/clergy	9	0.7	1.5
From conversations with colleagues	206	15.2	33.4
From conversations with migrant friends	63	4.6	10.2
From conversations with neighbors	23	1.7	3.7
From conversations with Polish friends	181	13.3	29.3
From conversations with the employer	216	15.9	35.0
From information from the Ukrainian minority organization	25	1.8	4.1
From talking to family members	88	6.5	14.3
From the page on Facebook	208	15.3	33.7
From information leaflets	179	13.2	29.0
From NGO information	54	4.0	8.8
Other source	106	7.8	17.2
Total	1358	100.0	220.1

Most respondents believe that the pandemic has not changed attitudes of Poles towards Ukrainians (69.5%) or attitudes of employers towards the respondent (56.4%). However, only 8.9% of respondents said that the pandemic has helped Ukrainians in Poland help each other more (see Tables 8–10).

**Table 8:** Opinions on how the pandemic has changed the behavior of Poles towards Ukrainians.

	Freq (n)	%
Poles' attitude towards Ukrainians improved	13	2.1
The attitude of Poles towards Ukrainians has not changed - Poles are friendly towards us	429	69.5
The attitude of Poles towards Ukrainians has not changed - Poles are hostile towards us	118	19.1
The attitude of Poles towards Ukrainians has worsened	57	9.2
Total	617	100.0

**Table 9:** Opinion on how the pandemic has changed your employer's behavior towards respondent.

	Freq (n)	%
The employer's attitude towards me has improved	35	5.7
The employer's attitude towards me has not changed - the employer has always helped me	348	56.4
The employer's attitude towards me has not changed - the employer has not helped me before	194	31.4
The employer's attitude towards me worsened	40	6.5
Total	617	100.0

**Table 10:** Respondents’ answers on question if the pandemic has made Ukrainians in Poland help each other more.

	Freq (n)	%
Yes	55	8.9
No	236	38.2
Hard to say	326	52.8
Total	617	100.0

## 2 Experimental Design, Materials and Methods

The situation of migrant workers in pandemic conditions is specific [1], [2] and includes issues such as cut-off of return/limitation of mobility, overcoming language barriers to accessing safety information, changes in the labour market (layoffs, bankruptcies, work stoppages).

The questionnaire was referring to these issues and developed on the basis of studies on migrant workers' opinions regarding crisis situations and changes in living conditions in the host country [1], [2], [3], [4].

The questionnaire contained 34 questions (in three languages: Ukrainian, Polish and Russian), including attribute variables of the participants). Questions addressed issues such as perception of COVID risks, changes in the respondent's labor market situation, and assessment of their quality-of-life changes.

The survey was conducted in May and June 2020 using an online form. The participants were 617 Ukrainian migrant workers who have remained in Poland during first COVID-19 lockdown. Participation was voluntary and participants were allowed to refuse or withdraw from the survey at any time.

Only the completed questionnaires were included in the dataset. The questionnaire (pdf attached) and raw data were translated into English. Data were processed anonymously; For descriptive statistics Excel file was imported to IBM SPSS 26.

## Ethics Statement

This manuscript has not been published elsewhere or it is not under consideration for publication for other journals. Participants were fully instructed about the survey aim and requirements and were informed that by agreeing to fill in the web questionnaire they confirmed their participation, automatically providing an informed consent. The Commission for Ethics for Social Science Research of University of Szczecin Institute confirmed that in accordance with the national legislation appropriate informed consent was obtained (approval number BN/01/03/2020). The survey did not collect any identifiable information from the participants.

## CRediT authorship contribution statement

**Dorota Kowalewska:** Conceptualization, Methodology, Writing – original draft. **Anita Adamczyk:** Conceptualization, Supervision, Investigation, Writing – original draft. **Monika Trojanowska–Strzęboszewska:** Investigation, Validation, Writing – original draft.

## Declaration of Competing Interest

The authors declare that they have no known competing financial interests or personal relationships which have or could be perceived to have influenced the work reported in this article.

## Appendix Supplementary materials

Image, application 1
