# Supplementary material for: Dataset of Ukrainian migrant workers opinions on their stay in Poland during COVID-19 lockdown
Source: Data Brief. 2021 Sep 22;38:107415. doi: 10.1016/j.dib.2021.107415 (PMC8457640; doi:10.1016/j.dib.2021.107415)
Supplement: Supplementary file 1 [file mmc1.pdf]

UKRAINIANS IN POLAND DURING THE PANDEMIC

The situation we find ourselves in with regard to the Covid-19 pandemic is a challenging experience. From mid-March 2020, restrictions have been introduced in relation to the epidemiological threat. We are keen to identify the difficulties faced by Ukrainian citizens who have stayed in Poland.

We would like to ask you to fill in the questionnaire. It is anonymous and will take a few minutes to complete.

The research is conducted by employees of the Institute of Political Science and Security at the University of Szczecin, the Faculty of Political Science and Journalism at the A. Mickiewicz University and the Institute of Political Science and Administration at the Cardinal Stefan Wyszyński University in Warsaw.

*Required

1/ Do you think that staying in Poland during the pandemic was a good choice? *

- ⇒ Definitely not
- ⇒ Probably not
- ⇒ Neither yes nor no
- ⇒ Probably yes
- ⇒ Definitely yes
- ⇒ N/A

2/ What do you fear in relation to the pandemic (you can give more than one answer) *

- ⇒ Covid19 of my relatives/friends
- ⇒ Deterioration of living conditions
- ⇒ I have no concerns about the pandemic
- ⇒ I would get Covid
- ⇒ It will not be possible to go to Ukraine
- ⇒ Loss of a job
- ⇒ Reduced salary
- ⇒ The deepening economic crisis

3/ How did your material status in time of Covid change: *

- ⇒ Got worse
- ⇒ It remained unchanged
- ⇒ Got better

4/ Why your economic situation has worsened(you can give more than one answer):*

- ⇒ I lost my job
- ⇒ Increased food prices
- ⇒ Increased rental prices (apartment)
- ⇒ loss of employment of a family member residing in Poland
- ⇒ Reduction of wages
- ⇒ Other:

5/ Will your staying in Poland during the pandemic have an impact on contacts with your family in Ukraine?*

- ⇒ Yes
- ⇒ No
- ⇒ I have no family in Ukraine

6/ Why did you choose to stay in Poland during the pandemic: (you can choose more than one answer): *

⇒ I did not know when it will be possible to return to Poland
⇒ I have tied my life plans with Poland and I do not intend to leave here
⇒ I was afraid (in general)
⇒ I was afraid of a compulsory quarantine in Ukraine
⇒ I was afraid that the situation in Ukraine during the pandemic would be worse than in Poland
⇒ Because I'm studying (in Poland)
⇒ Because of the job I have
⇒ Due to better medical care in Poland
⇒ Due to higher earnings in Poland
⇒ Due to the children attending school (in Poland)
⇒ Due to the situation of a family member (other than work)
⇒ Due to the work of a family member in Poland
⇒ For fear of forced quarantine in Ukraine
⇒ Other:

7/ Do you think that the decision of the Polish government to close the border was right?*

⇒ Definitely not
⇒ Probably not
⇒ Neither yes nor no
⇒ Probably yes
⇒ Definitely yes
⇒ I have no opinion

8/ How did you hear about the measures during a pandemic (you can provide more than one answer)*

⇒ From conversations with priests/clergy
⇒ From conversations with colleagues
⇒ From conversations with migrant friends
⇒ From conversations with neighbors
⇒ From conversations with Polish friends
⇒ From conversations with the employer
⇒ From information from the Ukrainian minority organization
⇒ From NGO information
⇒ From information leaflets
⇒ From talking to family members
⇒ From the page on fb
⇒ Other source

9/ Are you currently employed in Poland? *

⇒ Yes
⇒ No

10/ Please indicate how the period of the pandemic affected your labor market situation *.

⇒ I am dependent on my relatives / family
⇒ I am looking for a job
⇒ I am on compulsory unpaid leave
⇒ I am working but my working hours have been shortened
⇒ I receive unemployment benefits
⇒ I was dismissed due to pandemic

⇒ I work in a 'grey zone'
⇒ I'm not looking for a (new) job
⇒ I'm on a compulsory paid vacation
⇒ My salary went down
⇒ The terms of my employment have not changed

11/ Did your legal stay expire during the pandemic?*
⇒ Yes
⇒ No

12/ Did your employment permission expire during the pandemic? *
⇒ Yes
⇒ No

13/ Did you deal with official matters in Poland during the pandemic? *
⇒ Yes
⇒ No

14/ What were the official matters handled by you *
⇒ Employments
⇒ Family matters (e.g. related to children living in Poland)
⇒ Matters relating to registration of residence
⇒ Registration matters
⇒ Taxes
⇒ Vehicle registration
⇒ Other :

15/ Before the pandemic, did you receive any social assistance benefits? *
⇒ Yes
⇒ No

16/ If yes, please specify what type of benefit it was: ……………… [open question]

17/ Did the pandemic make you applying for social care benefits?? *
⇒ Yes
⇒ No

18/ If yes, please write what assistance you have requested? ……………..

19/ In your opinion, how the pandemic has changed the behavior of Poles towards Ukrainians?*
⇒ Poles' attitude towards Ukrainians improved
⇒ The attitude of Poles towards Ukrainians has not changed - Poles are friendly towards us
⇒ The attitude of Poles towards Ukrainians has not changed - Poles are hostile towards us
⇒ The attitude of Poles towards Ukrainians has worsened

20/ Do you think that the pandemic has changed your employer's behavior towards you? *
⇒ The employer's attitude towards me has improved
⇒ The employer's attitude towards me has not changed - the employer has always helped me
⇒ The employer's attitude towards me has not changed - the employer has not helped me before
⇒ The employer's attitude towards me worsened

21/ Do you think that the pandemic has made Ukrainians in Poland help each other more?

⇒ Yes
⇒ No
⇒ Hard to say

22/ Please answer, what is this mutual support? (open question).............................

23/ Do you have any children who attend school in Poland?? *

⇒ Yes
⇒ No
⇒ I have no children at all

24/ Was distance (online) learning during the pandemic a problem for your family?? *

⇒ Yes
⇒ No

25/ Did the family receive a computer / laptop / tablet during the pandemic?

⇒ No, because I don't know who to turn to for help
⇒ No, because we had our own computer equipment that met our needs
⇒ Yes, received from a non-governmental organization
⇒ Yes, received from Polish acquaintances
⇒ Yes, received from school

26/ Has the teacher at the school your child / children attend / were interested in the family situation during the pandemic? (you can choose more than one answer):*

⇒ I do not know
⇒ No, no one was interested in our situation
⇒ Yes, the class teacher is in contact with us
⇒ Yes, we are contacted by an assistant

Now please give us some information about yourself:

I1/Gender *

⇒ Woman
⇒ Man

I2/ Year of birth (YYYY): (please fill)

I3/ Length of stay in Poland *

⇒ Up to 4 months
⇒ From 4 to 6 months
⇒ More than 6 months but less than 1 year
⇒ 1-2 years (also with breaks)
⇒ 2-5 years (also with breaks)
⇒ More than 5 years (also with breaks)

I4/ Your residence status in Poland:

⇒ A temporary residence permit for the purpose of performing work by a foreigner delegated by a foreign employer to work in Poland
⇒ A temporary residence permit for the purpose of performing work requiring high qualifications
⇒ I do not know
⇒ Long-term resident's EU residence permit
⇒ Permanent residence permit

⇒ Temporary residence and work permit
⇒ Temporary residence permit
⇒ Temporary residence permit for family members of Polish citizens and family members of foreigners
⇒ Temporary residence permit for the purpose of performing work for the purpose of conducting business activity
⇒ Temporary residence permit in order to perform work in order to study at a university

I5/ Number of persons forming your household in Poland *

⇒ I live alone
⇒ 2 persons
⇒ 3 persons
⇒ 4 persons
⇒ 5 persons
⇒ More than 5 persons

I6/ Your place of residence in Poland *

⇒ A village away from the big city
⇒ A village near a big city
⇒ City up to 10,000 residents
⇒ City from 10,000 up to 100,000 residents
⇒ City from 100,000 to 500,000 residents
⇒ City over 500,000 residents

I7/ Please tick the province where you are staying at the moment: *

⇒ Dolnośląskie
⇒ Kujawsko-Pomorskie
⇒ Lubelskie
⇒ Lubuskie
⇒ Łódzkie
⇒ Małopolskie
⇒ Mazowieckie
⇒ Opolskie
⇒ Podkarpackie
⇒ Podlaskie
⇒ Pomorskie
⇒ Śląskie
⇒ Świętokrzyskie
⇒ Warminsko-Mazurskie
⇒ Wielkopolskie
⇒ Zachodniopomorskie

Thank you very much!
Any comments on the survey can be sent to:
dorota.kowalewska@usz.edu.pl
